# *B*-factor accuracy in protein crystal structures

**DOI:** 10.1107/S2059798321011736

**Published:** 2022-01-01

**Authors:** Oliviero Carugo

**Affiliations:** a Department of Chemistry, University of Pavia, Viale Taramelli 12, I-27100 Pavia, Italy; b Department of Structural and Computational Biology, University of Vienna, Campus Vienna Biocenter 5, A-1030 Vienna, Austria

**Keywords:** accuracy, *B* factors, normal probability plot, validation, protein crystal structures

## Abstract

The accuracy of *B* factors in protein crystal structures has been determined by comparing the same atoms in numerous, independent crystal structures of *Gallus gallus* lysozyme. Both *B*-factor absolute differences and normal probability plots indicate that the estimated *B*-factor errors are quite large, close to 9 Å^2^ in ambient-temperature structures and to 6 Å^2^ in low-temperature structures, and surprisingly are comparable to values estimated two decades ago. It is well known that *B* factors are not due to local movements only but reflect several, additional factors from crystal defects, large-scale disorder, diffraction data quality *etc*. It therefore remains essential to normalize *B* factors when comparing different crystal structures, although it has clearly been shown that they provide useful information about protein dynamics. Improved, quantitative analyses of raw *B* factors require novel experimental and computational tools that are able to disaggregate local movements from other features and properties that affect *B* factors.

## Abstract 2

The accuracy of *B* factors, estimated by comparing the same atoms in numerous protein crystal structures, is rather modest: close to 9 Å^2^ in ambient-temperature structures and to 6 Å^2^ in low-temperature structures. These values are similar to those estimated two decades ago, indicating that little has changed since.

## 1. Introduction

Data quality is essential in science, and during the last three decades structural biologists have developed several validation protocols aimed at minimizing pitfalls in their results and highlighting possible problems in their structures. Amongst the first validation tools, the importance of *PROCHECK* (Morris *et al.*, 1992 ▸; Laskowski *et al.*, 1993 ▸) cannot be underestimated. Its publication has been cited more than 21 000 times and nearly 700 times in 2021 (Google Scholar). The basic idea behind *PROCHECK* is that infrequent structural features, such as for example an anomalous φ/ψ position in the Ramchandran plot (Carugo & Djinović-Carugo, 2013 ▸), must be examined carefully. Although they might be genuine and suggesting something interesting, it is more probable that they are mistakes due to poor diffraction data quality or data-processing inaccuracy *etc*.

Several other validation tools have been developed (Read *et al.*, 2011 ▸). They included additional structural features, such as the positions of C^α^-bound H atoms in *MolProbity* (Chen *et al.*, 2010 ▸), which can be considered to be a sort of gold standard in today’s protein 3D model validation, or they address structures solved using a particular experimental method, such as *PROCHECK-NMR* (Laskowski *et al.*, 1996 ▸), which is dedicated to solution NMR structures. The Worldwide Protein Data Bank (wwPDB) then developed an integrated validation report for protein structures determined using several experimental methods (https://validate-rcsb-2.wwpdb.org/), which became the mandatory quality check for data deposited in the Protein Data Bank (PDB).

*B* factors have received less attention than other structural parameters for protein structure validation.

Recently, Murshudov and coworkers developed a computational technique that points out protein structures characterized by anomalous *B* factors (Dall’Antonia *et al.*, 2012 ▸; Masmaliyeva & Murshudov, 2019 ▸). Since the *B*-factor distribution in a protein crystal structure can be described with a shifted inverse-gamma function, 

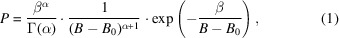

each structure is associated with parameters α (the function shape) and β (the scale). All proteins tend to cluster in a small region of the α versus β plot and outliers can be detected much like the φ/ψ outliers in a Ramachandran plot. Note that this approach is applicable to proteins where the distribution of the *B* factors is unimodal, which is typically the case for single and small domains. An extension of this computational method when the *B*-factor distribution is more complex is also available (Masmaliyeva *et al.*, 2020 ▸).

A relatively similar approach has been designed by Garman and coworkers (Gerstel *et al.*, 2015 ▸; Shelley *et al.*, 2018 ▸). Originally aimed to identify atoms for which the occupancy had decreased because of radiation damage during diffraction data collection, it is based on comparison of the *B* factor of an atom with the average *B* factor of all of the atoms that have the same packing density. Clearly, an anomalous *B* factor might be a sign of erroneous refinement and can highlight outliers that deserve further attention.

A different approach has been followed in two recent, additional studies. In the first study, the average *B* factors of protein crystal structures are related to the percentage of the cell volume occupied by water. By extrapolating the *B* factor corresponding to a hypothetical crystal that contains only water, it is possible to estimate the maximal *B* factor that is observable in protein crystals as a function of the crystallo­graphic resolution (Carugo, 2018*b* ▸). In the second study, atomic *B* factors are related to atomic solvent-accessible surface areas and the *B*-factor value associated with an atom that is completely solvent-accessible (isolated from any other atom) is extrapolated. In this way, it is possible to estimate, as a function of crystallographic resolution, the maximal *B* factor that individual atoms can show in protein crystal structures (Carugo, 2019 ▸).

Anisotropic *B* factors have also been examined. Zucker and coworkers designed methods for validating anisotropic *B* factors determined at atomic resolution and *B* factors derived from TLS (translation/libration/screw) refinements (Zucker *et al.*, 2010 ▸), and a server is available for this type of validation (http://skuld.bmsc.washington.edu/parvati). Merritt compared isotropic *B* factors (*B*_iso_) with their equivalent in structures refined anisotropically (*B*_eq_) and observed that they may differ, especially for atoms that are more anisotropic (Merritt, 2011 ▸). Criteria for choosing alternative treatments of *B*-factor refinements, depending on the amount of experimental information, have been defined (Merritt, 2012 ▸), and recently Afonine and coworkers designed a procedure to identify problematic TLS refinements (Afonine *et al.*, 2018 ▸).

This recent increase in interest in *B*-factor validation tools is clearly due to the increasing interest in the applications of *B* factors in molecular biology and in biotechnology (Carugo, 2018*a* ▸; Sun *et al.*, 2019 ▸).

Nevertheless, a crucial issue regarding *B* factors in protein crystal structures is their accuracy. Their estimated errors cannot in general be computed because of the paucity of diffraction data. It is, however, well known that *B* factors are poorly reproducible and can differ amongst different crystal structures (Ringe & Petsko, 1986 ▸). Of course, they depend markedly on the crystallographic resolution, but normalization of their values is a common routine when the *B* factors of different structures must be compared (Ringe & Petsko, 1986 ▸; Vihinen *et al.*, 1994 ▸; Carugo & Argos, 1997 ▸).

An attempt to estimate *B*-factor accuracy was published more than 20 years ago (Carugo & Argos, 1999 ▸) based on an analysis of ambient-temperature crystal structures; it was the norm at that time to collect diffraction data at room temperature. The PDB was much smaller than it is now; it contained fewer than 10 000 entries compared with the current 180 000 and the 15 000 that were released in 2020 alone. Moreover, data collection at low temperature, usually around 100 K, became the rule in macromolecular crystallography in order to protect samples from radiation damage, which is particularly intense when using the high flux densities of modern synchrotron X-ray sources (Carugo & Djinović-Carugo, 2005 ▸; Gerstel *et al.*, 2015 ▸; Garman & Owen, 2006 ▸).

Here, *B*-factor accuracy is determined by using a well controlled data set of protein crystal structures deposited in the PDB. More than 400 crystal structures of wild-type *Gallus gallus* lysozyme (space group *P*4_3_2_1_2) were downloaded from the PDB, about one third of which were determined at ambient temperature and the rest of which were determined at ∼100 K. Assuming that the *B* factors must be the same for the same atom in different structures, it is possible to estimate the degree of variability amongst different structures, obviously only comparing structures determined at similar crystallo­graphic resolution. Comparisons were performed by analyzing the absolute values of the differences between *B* factors of the same atom and by using normal probability plots.

## 2. Materials and methods

### 2.1. Data selection

It is well known that the PDB contains multiple structures of the same protein. It is mandatory in many data-mining processes to reduce this redundancy, which, on the other hand, may be beneficial in other applications. Here, the abundance of structures of the same protein is exploited in order to compare *B*-factor values in different structures of the same protein.

The crystal structure of lysozyme has been determined several times by different crystallographers in different laboratories: 429 crystal structures of wild-type *G. gallus* lysozyme were found in the PDB, according to the following criteria.

(i) Only crystal structures determined in space group *P*4_3_2_1_2 were retained.
(ii) PDB files containing only C^α^ atoms were removed.
(iii) PDB files containing nucleic acids in addition to lysozyme were removed.
(iv) PDB files containing too many heteroatoms (>5%; not including waters) were removed.
(v) Only single-model structures were retained.
(vi) Structures with average *B* factors larger than the maximal acceptable value (Carugo, 2018*b* ▸), which depends on the resolution, were rejected.
(vii) Atoms with abnormally large *B* factors (Carugo, 2019 ▸) were not considered.

156 of the 429 crystal structures were determined in the 280–300 K temperature range and the other 273 in the 90–110 K range. The crystallographic resolution ranges from 1.12 to 2.50 Å in the ambient-temperature structures [mean value of 1.79 (2) Å] and from 1.00 to 2.51 Å in the low-temperature structures [mean value of 1.58 (2) Å] (see Supplementary Fig. S1). The identification codes of all the PDB structures examined in the present communication are reported in Supplementary Table S1.

### 2.2. Delta values

The absolute values of the differences between the *B* factors of the same atom *A* in two structures *X* and *Y* were computed as

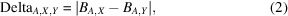

where *B*_*A*,*X*_ and *B*_*A*,*Y*_ are the *B* factors of atom *A* in structure *X* and of atom *A* in structure *Y*, respectively.

### 2.3. Normal probability plots

Normal probability plots (NPPs) are used to compare two sets of experimental data (**X** and **Y**) containing *n* variables. The difference *d_i_* between the two *i*th (1 ≤ *i* ≤ *n*) variables *x_i_* and *y_i_* is computed as

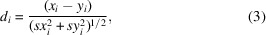

where *sx_i_* and *sy_i_* are the standard errors of *x_i_* and *y_i_*, respectively. The *d* values, sorted in order of increasing amplitude, are plotted versus the expected *de* values, which can be computed as 

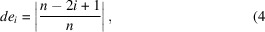

with their signs being positive if *i* > *n*/2 and negative if *i* < *n*/2. If the *n* points are fitted by a regression line with zero intercept and unit slope, **X** = **Y**. Otherwise, one can conclude either that **X** ≠ **Y** or that the standard errors *sx* and *sy* are underestimated. According to the second hypothesis, NPPs can be used to estimate the standard errors of the data. In this case, the observed differences *d* are plotted against their expected values *de* and the slope *a* of the regression line (*d* = *ade*) is used to estimate the average standard error sigma of the *B* factors as

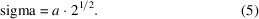

**Figure 1:**
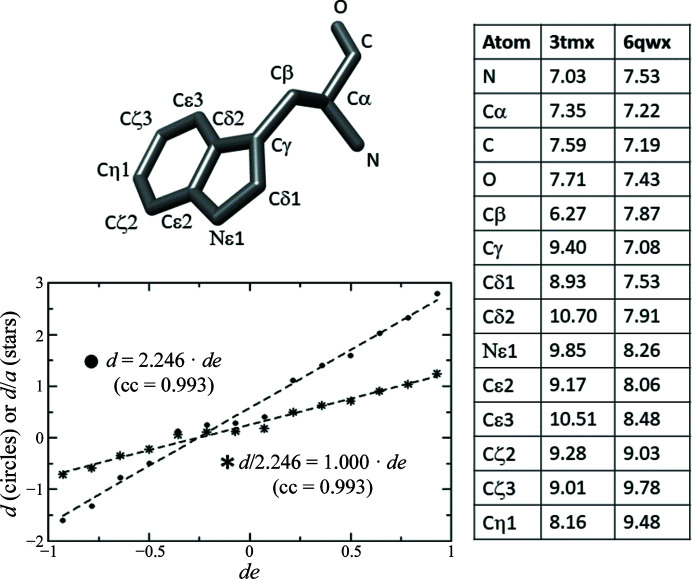
Example of a normal probability plot. The *B* factors of the 14 atoms of Trp28 in PDB entries 3tmx and 6qwx, shown on the right in Å^2^, allow a plot of *d* versus *de* (circles) to be made, which has a slope *a* of 2.246. Obviously, the of plot *d*/*a* versus *de* (stars) has a slope *a* of 1 and implies an average standard error of 1.59 Å^2^ for the *B* factors.

An example of an NPP is shown in Fig. 1 ▸. The *B* factors of the Trp28 atoms in PDB entries 3tmx (Kmetko *et al.*, 2011 ▸) and 6qwx (A. S. Boikova, P. V. Sorovatovskii, Y. A. Dyakova, K. B. Ilina, I. P. Kuranova, V. A. Lazarenko, M. A. Marchenkova, Y. V. Pisarevsky, V. I. Timofeev & M. V. Kovalchuk, unpublished work) are considered. The scatter plot of *d* versus *de* can be fitted by a straight line of slope 2.246, which implies that the two sets of *B* factors can be considered to be identical if the average *B*-factor standard error is 1.59 Å^2^.

### 2.4. Miscellaneous

Solvent-accessible surface areas were computed with *NACCESS* (Hubbard & Thornton, 1993 ▸) and secondary structures were assigned with *STRIDE* (Heinig & Frishman, 2004 ▸) and simplified into helix (H; *STRIDE* codes H, I and G), strand (E; *STRIDE* codes E, B and b) and loop (L; *STRIDE* codes T and C). All other computations were performed with locally written software.

## 3. Results and discussion

Several million Delta values, computed by comparing all pairs of equivalent atoms using equation (2), were classified according to crystallographic resolution (Table 1 ▸). Analogously, sigma values were also computed using equation (5) (Table 2 ▸). They are the estimated standard errors of *B* factors, computed under the assumption that the *B* factors of the same atom in different structures must be the same. Moreover, both Deltas and sigmas were classified according to several features: backbone or side-chain atoms, solvent-accessible (SASA ≥ 5 Å^2^) or buried (SASA < 5 Å^2^) atoms, and atoms belonging to residues in helical, strand or loop secondary structures (see Section 2 for details of the secondary-structure classification; Table 1 ▸ and Table 2 ▸). Although unrelated in their definition, Deltas and sigmas are quite well correlated (see Supplementary Fig. S2), indicating that they monitor the same feature in different ways.

### 3.1. Raw data

Considering any type of atom and independently of the resolution, both Deltas and sigmas are quite large, close to 9 Å^2^ in ambient-temperature structures and to 6 Å^2^ in low-temperature structures. These values compare well with those estimated two decades ago (Carugo & Argos, 1999 ▸) and suggest, as a consequence, that the determination and refinement of *B* factors has not evolved much.

Similar values were observed in protein structure subsets assembled according to the software suite used for structure refinement.

These large values suggest that the accuracy of *B* factors in protein structures is actually quite limited and indicate that it can be hazardous to compare *B* factors of different crystal structures. It is, in other words, highly advisable to normalize them, for example to zero mean and unit variance.

This agrees with the results recently published by Pearce & Gros (2021 ▸), who designed an elegant decomposition of *B* factors into several components: one due to the entire protein, one associated with secondary-structural elements, one associated with individual residues, one split into the backbone and side chain of each residue and the last one due to local atomic motions. The last component, which describes the real positional spread of the atom due to local interactions, is actually quite modest and different from the refined global *B* factor. The weight of each component may be different amongst crystal structures and, as a consequence, different *B* factors can be observed.

Larger Deltas and sigmas are observed in ambient temperature structures than in low-temperature structures. This might have multiple explanations. On one hand it is possible that more reliable *B*-factor refinements are possible at low temperature, and on the other hand it is possible that the fact that *B* factors tend to be smaller at low temperature limits their variability. Moreover, one must consider that protein dynamics is influenced by temperature (Vitkup *et al.*, 2000 ▸; Ringe & Petsko, 2003 ▸) and it might be interesting to apply the procedure described by Pearce & Gros (2021 ▸) to structures of the same protein determined at different temperatures.

### 3.2. Disaggregated data

Deltas and sigmas are quite similar in different types of atoms (solvent accessible or not, backbone or side chain, or in different types of secondary structures; Tables 1 ▸ and 2 ▸).

Side-chain atoms show Deltas and sigmas that are slightly larger than those of backbone atoms both at ambient and at low temperature. In contrast, while the Deltas of solvent-accessible atoms are slightly larger than those of atoms buried in the protein core, the opposite is observed for sigmas, which are slightly larger in buried atoms than in solvent-accessible atoms both at low and at ambient temperature.

Atoms in loop residues have both Deltas and sigmas that are slightly larger than those of atoms in ordered secondary structures (helix and strand) both at ambient and low temperature.

However, all of these differences tend to be quite modest both at low and ambient temperature and marginal when compared with the differences due to the different temperature.

### 3.3. Resolution

Interestingly, the values of Delta and sigma are independent of resolution in the resolution range examined here (Tables 1 ▸ and 2 ▸). This is surprising, since it is reasonable to suppose that at higher resolution, thanks to a larger quantity of experimental information, better refinements of every structural variable are possible, with consequent convergence of the *B* factors towards a common value amongst different crystal structures. Furthermore, at higher resolution the crystal quality is expected to be better and, as a consequence, the components of the *B* factor due to nonlocal factors (Pearce & Gros, 2021 ▸) are expected to be smaller. The *B* factor should therefore reflect the genuine flexibility of the atoms.

On the contrary, Delta values vary randomly from higher to lower resolution. They range from 6.5 to 9.2 Å^2^ in ambient-temperature structures (1.8 and 2.1 Å resolution, respectively) and from 6.0 to 9.0 Å^2^ in low-temperature structures (2.2 and 1.6 Å resolution, respectively). Analogously, sigma values range from 7.7 to 9.2 Å^2^ in ambient-temperature structures (1.8 and 2.0 Å resolution, respectively) and from 6.7 to 9.3 Å^2^ in low-temperature structures (<1.5 and 2.1 Å resolution, respectively).

It can thus be hypothesized that the accuracy in refining *B* factors is limited not only by the amount of experimental information but by other factors, such as for example the quality of the data themselves, which is influenced by several factors (for example lattice defects, radiation damage *etc.*), or details in computational methods (for example scaling, lattice constant determination *etc.*).

This strongly supports the need to normalize *B* factors before comparing different protein crystal structures, even if they have been refined at high resolution (up to 1 Å in the set of structures examined in the present manuscript). Although it is not certain which kind of normalization is preferable (Carugo, 2018*a* ▸), it is certain that unless the resolution reaches the levels common in small-molecule crystallography (better than 0.8 Å resolution; there are no structures of this resolution in the data examined in the present manuscript), some normalization is required. Although *B* factors have proven to provide important and useful information to better understand molecular dynamics and design biotechnological applications (Sun *et al.*, 2019 ▸; Carugo, 2018*a* ▸), they cannot be used to quantitatively describe local atomic motion.

## 4. Conclusions

This manuscript analyzes the reproducibility of *B* factors in protein crystal structures and there is one main conclusion: they are not really reproducible, with estimated errors of about 9 and 6 Å^2^ in ambient- and low-temperature structures. Interestingly, the level of reproducibility seems to have remained unchanged over the last two decades (Carugo & Argos, 1999 ▸) and it does not depend on resolution, at least in the resolution range examined here.

This clearly indicates that *B* factors do not monitor only atomic motions but, as is well known, also other features, including crystal defects, diffraction decay, computational details *etc.*

An important diffraction feature is, for example, the mosaicity, which is due to crystal defects and may increase during data collection because of radiation damage, especially at ambient temperature. However, little information is deposited about mosaicity in the Protein Data Bank.

The chemical composition of the crystallization cocktail might be a relevant variable, since the presence of small molecules at the protein surface might influence the *B* factors of protein surface atoms. However, it is extremely difficult to obtain reliable information about the crystallization cocktail directly from the PDB files, since these annotations are not always present and, when present, are not always reported with the same accuracy.

In addition, the crystallization method might be an interesting feature to consider. This information is present in about 70% of the ambient-temperature structures and in about 80% of the low-temperature structures. However, only in the set of ambient-temperature structures it is possible to make a statistically sensible comparison between structures determined with vapor diffusion and batch crystallization (all of the other crystallization techniques are infrequent amongst the ambient-temperature structures, and the large majority of the low-temperature structures were obtained by vapor-diffusion crystallization, with few examples of other techniques). However, both the Delta and sigma values were nearly the same, independently of the crystallization method.

All of these considerations indicate that it is still mandatory to normalize *B* factors when comparing different crystal structure determinations, although it remains unclear whether a standardization method should be preferred over other methods (Carugo, 2018*a* ▸). This has been known for many years (Ringe & Petsko, 1986 ▸) and apparently little has changed since.

A recent publication might enlighten us about this problem. Pearce and Gros recently proposed a decomposition of the *B* factor into several components, thanks to a hierarchical TLS refinement strategy, which allows one to discover the fraction of the *B* factor that is really due to local positional fluctuations, independent of higher level factors (Pearce & Gros, 2021 ▸). In some selected examples, the *B*-factor component associated with local positional fluctuations, which is actually what should be conserved amongst different crystal structures, is quite small compared with the overall *B* factor (Pearce & Gros, 2021 ▸), which is determined by other factors that may differ amongst different crystal specimens and different structure determinations.

These results should prove to be useful to improve and strengthen the analysis and understanding of protein dynamics based on solid-state experimental findings.

## Supplementary Material

Supplementary Table and Figures. DOI: 10.1107/S2059798321011736/ni5016sup1.pdf

## Figures and Tables

**Table:** Data are shown only if there are at least 2000 observations. Estimated standard deviations, which in most of the cases are <0.01 Å^2^, are not reported explicitly for the sake of simplicity.

	any		mc		sc	
res	amb	low	amb	low	amb	low
<1.55	6.80	6.14	6.49	7.27	7.14	7.56
1.55–1.65	7.98	8.98	7.71	8.89	8.22	9.23
1.65–1.75	8.77	6.26	8.19	5.97	8.23	6.52
1.75–1.85	6.49	6.54	6.07	6.85	6.34	7.11
1.85–1.95	6.93	6.46	6.67	6.24	7.21	6.70
1.95–2.05	8.23	8.89	7.97	8.89	8.52	8.81
2.05–2.15	9.25	8.35	9.16	8.24	9.62	8.49
2.15–2.25	—	6.03	—	5.83	—	6.25
2.25–2.35	6.88	—	6.89	—	6.85	—
All	8.83	6.29	7.96	6.64	8.25	7.18

**Table:** 

	acc		bur	
res	amb	low	amb	low
<1.55	7.28	6.99	6.58	5.78
1.55–1.65	8.65	9.48	7.68	8.77
1.65–1.75	8.92	6.89	8.69	5.99
1.75–1.85	7.03	7.07	6.25	6.32
1.85–1.95	7.62	7.24	6.62	6.13
1.95–2.05	8.87	9.25	7.96	8.73
2.05–2.15	9.59	9.15	9.11	8.03
2.15–2.25	—	6.74	—	5.73
2.25–2.35	6.85	—	6.88	—
All	8.97	6.85	8.77	6.04

**Table:** 

	H		E		L	
res	amb	low	amb	low	amb	low
<1.55	6.81	5.79	6.72	5.90	6.80	6.57
1.55–1.65	7.87	8.92	7.72	8.43	8.14	9.15
1.65–1.75	8.68	6.01	8.72	5.97	8.85	6.59
1.75–1.85	6.09	6.39	6.18	5.99	6.94	6.83
1.85–1.95	6.73	6.35	6.62	5.63	7.19	6.75
1.95–2.05	8.03	8.70	8.04	8.54	8.48	9.16
2.05–2.15	9.02	8.45	9.11	7.01	9.52	8.55
2.15–2.25	—	5.76	—	—	—	6.47
2.25–2.35	6.81	—	—	—	6.99	—
All	8.60	6.20	8.90	5.88	9.02	6.48

**Table:** Data are shown only if there are at least 2000 observations.

	any		mc		sc	
res	amb	low	amb	low	amb	low
<1.55	8.47	6.66	8.25	6.78	8.72	7.32
1.55–1.65	9.16	9.27	9.03	9.24	9.27	9.46
1.65–1.75	8.78	7.99	8.55	7.62	8.74	7.96
1.75–1.85	7.70	8.00	7.44	7.92	7.64	8.17
1.85–1.95	8.45	7.90	8.36	7.80	8.55	8.01
1.95–2.05	9.20	9.60	9.13	9.62	9.28	9.53
2.05–2.15	8.97	9.30	8.91	9.24	9.11	9.36
2.15–2.25	—	6.84	—	6.67	—	7.02
2.25–2.35	5.57	—	5.36	—	5.78	—
All	9.01	6.57	8.69	6.10	8.85	6.90

**Table:** 

	acc		bur	
res	amb	low	amb	low
<1.55	8.33	6.26	8.78	8.57
1.55–1.65	9.02	9.24	9.49	9.58
1.65–1.75	8.51	7.84	8.94	8.19
1.75–1.85	7.38	8.12	7.90	8.31
1.85–1.95	8.28	7.74	8.82	8.29
1.95–2.05	9.13	9.56	9.37	9.70
2.05–2.15	8.90	9.17	9.12	9.60
2.15–2.25	—	6.65	—	7.26
2.25–2.35	5.36	—	6.02	—
All	8.81	6.11	8.99	7.70

**Table:** 

	H		E		C	
res	amb	low	amb	low	amb	low
<1.55	8.49	6.91	8.38	6.66	8.47	7.46
1.55–1.65	9.09	9.29	9.05	9.19	9.26	9.42
1.65–1.75	8.60	7.66	8.50	7.44	8.71	7.98
1.75–1.85	7.38	7.90	7.31	7.84	7.74	8.24
1.85–1.95	8.37	7.82	8.20	7.47	8.58	8.07
1.95–2.05	9.17	9.55	9.10	9.45	9.25	9.69
2.05–2.15	8.96	9.34	8.80	8.64	9.02	9.40
2.15–2.25	—	6.80	—	5.75	—	7.09
2.25–2.35	5.63	—	5.05	—	5.62	—
All	8.91	6.04	9.03	6.27	8.93	7.01
