# Supplementary material for: B-factor accuracy in protein crystal structures
Source: Acta Crystallogr D Struct Biol. 2022 Jan 1;78(Pt 1):69–74. doi: 10.1107/S2059798321011736 (PMC8725162; doi:10.1107/S2059798321011736)

STRUCTURAL BIOLOGY

Volume 78 (2022)

Supporting information for article:

B-factor accuracy in protein crystal structures

Oliviero Carugo

Table S1 List of the PDB entries examined in the present study.

Ambient temperature structures (280-300 K)

7byp 3tmv 4wl7 6jxp 7d05 7d01 7bvo 7dtf 7cvj 7byo 6ll3 3tmu 7dtb 6kd1 7e02 7d04 7d02 7cvl 4neb 4et8 5dm9 5njs 3wxt 7cdq 1rfp 4yen 6rxi 4xjh 1ykz 5apd 4g4b 7cdo 4z98 3wum 6k8g 4nfv 4ym8 4xjf 5uvj 4lyo 4wly 1yl0 1hel 5apf 6qqf 7cdm 6qy0 4ng1 4g49 6qq3 7cdk 4xjb 4rw2 6rnc 4wm5 4wlt 1lyo 1b0d 6ftr 4xn6 6qy4 1azf 1jiy 4wm3 6qy2 1uih 4etd 1bwj 4wm1 6h0k 6qxw 7cdu 1bvx 5la5 1yky 6q88 1z55 4etb 6jzi 1vdt 5njm 4ngl 7cds 6muz 6apm 2yvb 3wxu 1jis 7cdp 4ngv 4et9 5apc 3n9e 5kkj 5njr 1bwh 7cdn 2lyo 5wra 3wun 2cds 5l9j 6qxy 5ape 4g4c 1jj0 6qy1 3wul 4nwh 4rw1 5wr9 3n9a 1yl1 6ybi 5fel 7l84 4wlx 3n9c 6qy5 3zek 1uig 4wld 6rnb 4wmg 4eta 4nwe 4ngo 6qnb 6tvy 6ybf 5lan 1uc0 5mjj 4wm4 4rln 5ne0 6qxx 7cdt 1ykx 5f81 4ete 1bwi 5wrb 4wm2 6h0l 4ngk 6gf0 7cdr 4yop 3lyo 4etc 1jit 4bs7 2epe 1vds 3m3u

---

Low temperature structures (90-110 K)

3tmx 3b6l 3b72 3tmw 2bpu 5t3f 2c8o 4ppo 6w7p 5kki 6g5y 6agn 4nsg 4h8y 5b59 4dd3 5kxm 2h9j 4ngw 1vat 4nsi 6pl9 4nij 4h9i 2ybj 5laf 6qwy 2x0a 4n9r 4p2e 5d5f 6yoc 3t6u 2ybh 6a4o 5ebh 1dpx 4nhi 5k2s 5kxk 2xth 3a91 3ru5 2ybl 6qqd 3ijv 3txe 5b06 5ob8 5ilc 3agg 4xjd 6qea 4xad 4nht 5ob6 2ybn 5fst 5kj9 6ciw 4h1p 6yjx 4lyb 1yik 6q8t 2i6z 3atn 5iel 5i54 5ilf 2zyp 4ii8 4owb 6set 4b4e 4htk 6lt5 4h91 6abn 3a96 4dt3 3az6 4wo9 4h9b 2bly 6q8r 5dla 3agh 6pla 6goi 5fcp 6mx9 1gwd 6f1p 3a94 4hsf 4nhq 7d0w 5k2k 2xbs 5amy 6wrl 6sex 5i4w 5v4h 4c3w 3rz4 3sp3 4qeq 4hpi 4b4i 2w1m 6yoe 6f1r 4oot 4ngj 4h9f 4kxi 4hv1 5k2q 6mqv 6g5v 4uwv 6b7w 1w6z 5f9u 4ny5 4h93 5lin 1lz9 7der 4fjr 6b7u 4ooo 4woa 4lyc 5f9x 5la8 2hu1 4h8z 4xjg 5kxl 4qy9 2c8p 4axt 3ems 6rt3 3txj 6qwz 3w6a 1t3p 5dl9 6sez 4htq 6yob 6a4p 2ybi 4yem 2hu3 3a8z 5ihg 5k2r 2a7d 1dpw 4h8x 2cgi 6fsj 6rt1 4h9h 2d91 5lag 5gwb 6qwx 2hub 3iju 6ahl 5ob9 5b05 6a4n 1yil 6adf 6qx0 6g8a 1vau 3a92 4nhs 6ad5 5nbj 4x3b 5l3h 6qqe 6qqc 6ac2 5b07 5ob7 5kxn 4owh 6bo1 5ole 3a90 2ybm 2w1x 5yky 4h92 3a95 5hmj 2htx 3az7 2xbr 2w1y 6ahh 4h9c 4lfp 4j1b 3rnx 2blx 5ii3 3wmk 4owc 1qio 4qgz 4h90 4w96 3a93 3qng 6aea 4nhp 4wo6 5l3i 4gcb 4h9a 3ajn 5i53 4n8z 5e9r 3agi 6plb 6goj 4owe 2w1l 6yod 4w94 6wx5 4ng8 4b49 2a7f 5k2p 6qww 6wrm 2h9k 6sew 6abz 5i4x 5v4g 6agr 6syd 5lxw 4htn 7kh5 5lio 5d5c 4h9e 5k2n 4xji 6b7v 3ato 2ydg 5v4i 4zfp 4h94 6seu 4b0d 4b4j

---

Figure S1 Distributions of the resolution values in the set of ambient temperature structures (red) and in the set of low temperature structures (blue).

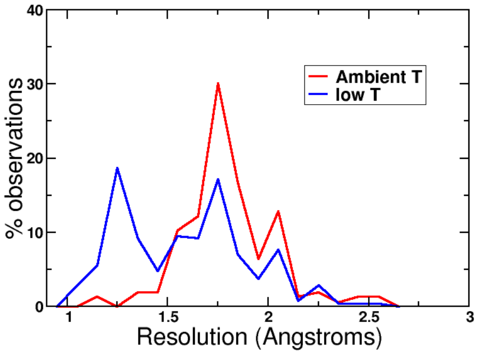

Figure S2 Relationship between *Delta* and *sigma* in ambient (red) and low (blue) crystal structures. Each point represents the mean value in a given resolution range (see **Tables 1 and 2**).

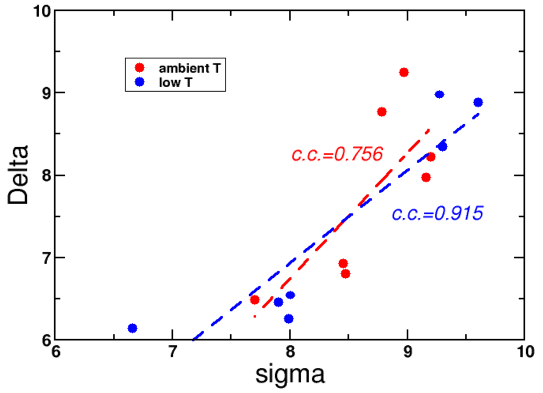

Supplement: Supplementary file 1 [file d-78-00069-sup1.pdf]
